# High-functioning autism spectrum disorder and fragile X syndrome: report of two affected sisters

**DOI:** 10.1186/2040-2392-3-5

**Published:** 2012-06-27

**Authors:** Pauline Chaste, Catalina Betancur, Marion Gérard-Blanluet, Anne Bargiacchi, Suzanne Kuzbari, Séverine Drunat, Marion Leboyer, Thomas Bourgeron, Richard Delorme

**Affiliations:** 1 Human Genetics and Cognitive Functions, Institut Pasteur, Paris, France; 2 INSERM, U955, Créteil, France; 3 APHP, Robert Debré Hospital, Department of Child and Adolescent Psychiatry, Paris, France; 4 INSERM, U952, Paris, France; 5 CNRS, UMR 7224, Paris, France; 6 UPMC, Université Paris 06, Paris, France; 7 APHP, Department of Genetics, Robert Debré Hospital, Paris, France; 8 APHP, Department of Adult Psychiatry, Henri Mondor-Albert Chenevier Hospitals, Créteil, France; 9 Faculté de Médecine, Université Paris-Est, Créteil, France; 10 CNRS, URA 2182, Paris, France; 11 Université Denis Diderot Paris 7, Paris, France

**Keywords:** Autism spectrum disorders, Female, Fragile X syndrome, Intellectual disability

## Abstract

**Background:**

Fragile X syndrome (FXS) is the most common inherited cause of intellectual disability (ID), as well as the most frequent monogenic cause of autism spectrum disorder (ASD). Men with FXS exhibit ID, often associated with autistics features, whereas women heterozygous for the full mutation are typically less severely affected; about half have a normal or borderline intelligence quotient (IQ). Previous findings have shown a strong association between ID and ASD in both men and women with FXS. We describe here the case of two sisters with ASD and FXS but without ID. One of the sisters presented with high-functioning autism, the other one with pervasive developmental disorder not otherwise specified and low normal IQ.

**Methods:**

The methylation status of the mutated *FMR1* alleles was examined by Southern blot and methylation-sensitive polymerase chain reaction. The X-chromosome inactivation was determined by analyzing the methylation status of the androgen receptor at Xq12.

**Results:**

Both sisters carried a full mutation in the *FMR1* gene, with complete methylation and random X chromosome inactivation. We present the phenotype of the two sisters and other family members.

**Conclusions:**

These findings suggest that autistic behaviors and cognitive impairment can manifest as independent traits in FXS. Mutations in *FMR1,* known to cause syndromic autism, may also contribute to the etiology of high-functioning, non-syndromic ASD, particularly in women. Thus, screening for FXS in patients with ASD should not be limited to those with comorbid ID.

## Background

Fragile X syndrome (FXS) is caused by the expansion of a CGG repeat in the 5′ untranslated region of the *FMR1* gene located at Xq27.3 (for review see [1]). In its normal form, *FMR1* contains 5 to 55 CGG repeats. In the presence of over 200 repeats there is extensive methylation of the CpG island in the gene’s promoter region, resulting in silencing of *FMR1* expression. Premutation alleles with 55 to 200 repeats are unstable and may expand to full mutation alleles when transmitted maternally. Fragile X mental retardation protein (FMRP), encoded by *FMR1*, is an RNA-binding protein that travels from the nucleus to the cytoplasm, where it regulates synaptic and cytoskeleton-associated proteins [2]. In the absence of FMRP, excessive and dysregulated mRNA translation leads to altered synaptic function and loss of protein synthesis-dependent plasticity [1]. FMRP can also regulate mRNA transport in dendrites and is important for axonal guidance and formation of neural circuits.

The cognitive, behavioral and morphological manifestations of FXS are highly variable [3]. Individuals with FXS often display mild dysmorphic features, with long face, prominent ears, arched palate and macroorchidism. They can also exhibit a large spectrum of neuropsychiatric phenotypes, spanning from severe intellectual disability (ID) to mild learning problems or emotional dysregulation, especially in heterozygous women [3]. Autistic behaviors, including impairments in social interaction, social anxiety, gaze avoidance, sensory hypersensitivity, stereotypic movements and behaviors, poor motor coordination, delayed speech development and echolalia, are also observed frequently in individuals with FXS [4]. FXS is the most common monogenic cause of autism spectrum disorders (ASD), identified in approximately 2% of cases [5,6]. Around 40% to 60% of male and 20% of female patients with FXS meet criteria for ASD [7-9]. Men with FXS often display both ID and autistic features, with those exhibiting autistic traits being more impaired [8-15]. In contrast, women are protected by the normal allele on the second X chromosome and usually exhibit a less severe phenotype. Although approximately 25% to 30% have ID (intelligence quotient (IQ) < 70), women with FXS usually have an IQ in the borderline to low normal range (75 to 90); most, however, exhibit neurocognitive dysfunctions and anxiety as well as emotional and attention deficits [16,17]. The variability of FXS-related phenotype in women is influenced by the level of residual FMRP expression and X inactivation skewing. Skewing that favors the mutant allele increases the clinical impairment, whereas skewing favoring the normal allele decreases the likelihood of symptoms [18,19]. In contrast with the frequent claim that cognitive impairment accounts for the co-morbidity between FXS and autism [13,20,21], here we describe the case of two sisters, one with high-functioning autism and the other with pervasive developmental disorder not otherwise specified (PDD-NOS), both with low normal IQ. Both carried a full mutation in the *FMR1* gene with random X inactivation.

## Methods

### Laboratory investigations

Genomic DNA was isolated from blood lymphocytes. The analysis of the *FMR1* gene was performed using standard methods: Southern blotting with *Eco*RI and *Eag*1 double digest and the StB12.3 probe, direct amplification of the CGG-repeat using flanking primers, and methylation-sensitive PCR after bisulphite treatment of DNA. The X-chromosome inactivation ratio was determined by analyzing the methylation status of the androgen receptor (*AR*) locus at Xq12, as previously described [22]. The X-inactivation ratio ranges from 50:50 (random inactivation) to 100:0 (complete non-random inactivation). X inactivation was considered skewed if the ratio was over 80:20.

To further delineate the contribution of rare genomic variants to the clinical phenotype, we genotyped the index case by using the Illumina Infinium 1 M single nucleotide polymorphism microarray (Illumina Inc, San Diego, CA). We used two copy number variation (CNV) calling algorithms, QuantiSNP and PennCNV, and the CNV viewer SnipPeep (http://snippeep.sourceforge.net/). To obtain high-confidence calls, the CNVs identified were validated by visual inspection of the Log R ratio and B allele frequency values. Criteria for detecting rare CNVs have been described previously [23].

### Ethical statement

This study was approved by the local institutional review board (Comité de Protection des Personnes Île-de-France VI, Hôpital Pitié-Salpêtrière). Written informed consent was obtained from all participants of the study, signed by the patients, the parents or the legal representatives.

## Results

### Clinical reports

***Patient 1:*** Thirty year-old female, referred to our clinic for assessment of high-functioning ASD. She is the first child of healthy non-consanguineous Caucasian parents. Pregnancy and delivery were normal. She was born at gestational week 39, and her weight (3,900 g), length (50 cm) and head circumference (35 cm) were in the normal range. Early development was considered normal despite moderate sleep difficulties and delay in walking (18 months). Her first words occurred before 24 months, but her parents reported an apparent stagnation of her development. When she started school at three years of age, her language was poorly developed and stereotyped. Teachers mentioned that she was isolated, lacked social reciprocity and had major misunderstandings of social cues. She had a small repertoire of facial expressions and limited use of gestures to communicate. She also exhibited various stereotyped behaviors associated with restricted interests. Despite her difficulty to integrate at school, she acquired normal writing and reading skills, and attended a normal school until 15 years of age. At this time, unable to follow a professional formation, she integrated a psychiatric day care two days per week for 9 years, and now remains at home. When referred to our center at the age of 30, she received a diagnosis of high-functioning autism based on Diagnostic and Statistical Manual of Mental Disorders, 4th Edition (DSM-IV) and International Statistical Classification of Diseases and Related Health Problems, 10th Revision (ICD-10) criteria. Assessment of ASD was performed using the Autism Diagnostic Interview-Revised (ADI-R) and the Autism Diagnostic Observation Schedule (ADOS); she met criteria for autism on both instruments (Table 1). Her IQ, measured with the Wechsler Adult Intelligence Scale (WAIS), was in the low normal range, with a full scale IQ of 85. Comorbid Axis I mental disorders were evaluated with the Diagnostic Interview for Genetic Studies (DIGS). The patient had no specific associated disorders, but fulfilled DSM-IV criteria for chronic motor tics. At examination, she had a long face, with a high forehead and normal ears. Her general and neurological examination was normal, with mild generalized obesity (body mass index 31, height 1.59 m, weight 78.4 kg). She had generalized hirsutism, related to a partial 21-hydroxylase deficiency discovered during late childhood. She had increased levels of 17-hydroxyprogesterone and adrenal androgens; molecular genetic testing was not performed.

**Table 1:** Autism Diagnostic Interview-Revised and Autism Diagnostic Observation Schedule scores in Patients 1 and 2

	**Patient 1**	**Patient 2**
**ADI-R**		
Age at evaluation (y)	30	28
Social (cut-off = 10)	20	11
Verbal communication (cut-off = 8)	17	4
Repetitive behavior (cut-off = 3)	6	1
Abnormality prior to 36 months (cut-off = 1)	3	1
ADI-R diagnosis	autism	not autism
**ADOS (module 4)**		
Age at evaluation (y)	33 yes	28
Communication (cut-off autism = 3, ASD = 2)	6	4
Social interaction (cut-off autism = 6, ASD = 4)	14	6
Total communication + social (cut-off autism = 10,ASD = 7)	20	10
Imagination	3	0
Restricted behaviors and interests	8	0
ADOS diagnosis	autism	autism
Final research diagnosis	autism	PDD NOS

*ADI-R* Autism Diagnostic Interview-Revised, *ADOS* Autism Diagnostic Observation Schedule, *ASD* autism spectrum disorder, *PDD-NOS* pervasive developmental disorder not otherwise specified.

***Patient 2:*** Twenty-eight-year-old female at the time of assessment, born at full-term after an uneventful pregnancy. Her motor and language milestones were normal. She integrated school early, but showed major difficulties in social interactions. She was isolated despite being interested in others. Similar to her sister, teachers mentioned difficulties in her relationships with schoolmates. At that time, parents and teachers considered that she only had symptoms of social avoidance. Despite a weakness in receptive and expressive language, she pursued a normal education until 18 years of age. She completed a professional formation and obtained a degree in childcare. She worked at different nurseries for short periods of time, but was unable to keep her job.

After the diagnosis of FXS and ASD in her sister, she was referred to our hospital for a clinical evaluation. Her prosody was normal, but her vocabulary was clearly limited. Direct eye gaze was rare and interaction difficult to establish. The clinical evaluation revealed that she had PDD-NOS. She did not reach cut-off for autism on the ADI-R, but met criteria for autism on the ADOS (Table 1). Evaluation with the WAIS-III showed a verbal IQ of 81 and a non-verbal IQ of 96. The patient had a lifetime history of panic disorder without agoraphobia. However, this diagnosis remained uncertain because a few months later, hyperthyroidism was discovered and treated, which resulted in remission of the panic disorder. Unlike her sister, she did not have a medical history of congenital adrenal hyperplasia. At examination, she had no dysmorphic features. Her general and neurological examination was normal except for mild generalized obesity (body mass index 32, height 1.59 m, weight 81.3 kg).

**Figure 1:**
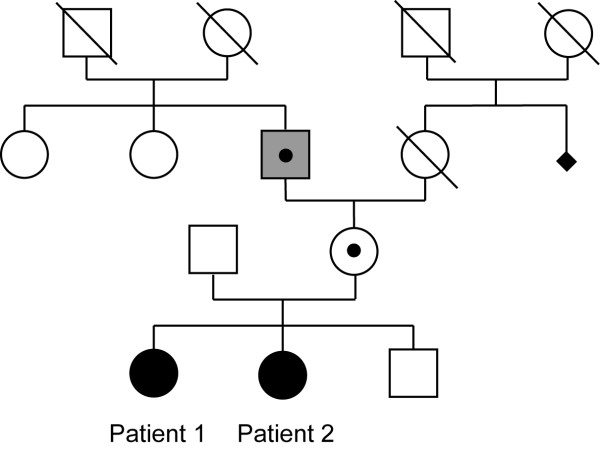
**Pedigree of the two affected sisters (black circles) with fragile X syndrome and high functioning autism spectrum disorder.** The mother and the paternal grandfather carried the premutation (black dots). The grandfather had fragile X associated tremor/ataxia syndrome (grey square). The black diamond indicates a miscarriage of indeterminate gender.

***Other members of the pedigree*** (Figure 1): A younger brother was reportedly healthy. The father did not report any personal or familial medical or psychiatric history. His clinical examination at the age of 58 years was within normal limits. The mother had no specific medical history, with the exception of panic disorder associated with agoraphobia from the age of 20 years. She did not report any sign of premature ovarian failure. When evaluated at the age of 57 years, her clinical examination was normal and she had a normal intelligence (WAIS III: verbal IQ 104, non verbal IQ 117, full scale IQ 110). Her father, who died at 85 years of age, had been hospitalized for dementia and ataxia. Clinical reports mentioned that he showed spasticity, tremor and rigidity as well as severe cognitive impairment, with onset around 75 years of age. The diagnosis of FXS in his granddaughter led to the re-assessment of his medical records and he was diagnosed retrospectively with fragile X-associated tremor/ataxia syndrome (FXTAS), which affects carriers, principally men, of premutation alleles of the *FMR1* gene. A review of brain biopsies obtained during autopsy revealed lesions typical of FXTAS, combining a few eosinophilic nuclear inclusions in the hippocampus and near the pons, affecting both neurons and glia, and enlarged inclusion-bearing astrocytes. In addition, neuropathological findings characteristic of Parkinson’s disease were observed in the locus coeruleus and substantia nigra. Numerous neurofibrillary tangles in the hippocampus and neuritic plaques in the isocortex suggested an associated Alzheimer disease, Braak stage 5.

### Analysis of the *FMR1* gene and X inactivation

Southern blot analysis of the CGG expansion at *FMR1* revealed a full mutation in both patients, with about 450 CGG repeats in the proband and 370 CGG repeats in her sister. Both mutated alleles were associated with complete methylation based on the Southern blot profile. The second allele was normal in the two sisters, with 29 repeats. The asymptomatic mother carried a premutated allele (93 repeats), as did the mother's father (100 repeats). In addition, no rare CNVs were detected in Patient 1, either on the X chromosome or the autosomes.

We hypothesized that the lack of intellectual deficit in the sisters could be linked to a skewed X inactivation in favor of the non-mutated allele. However, the X inactivation profile, assessed at the *AR* locus*,* was normal in the proband (45:55), her sister (47:53) and the mother (45:55).

## Discussion

Women who are heterozygous for the full *FMR1* mutation are less cognitively impaired than men, with about one-third presenting with ID and the rest exhibiting IQs in the borderline to low normal range [11,16,17]. Although the majority of women with FXS are not intellectually disabled, they often manifest a wide range of psychiatric and cognitive impairments, including anxious disorders, attention deficit-hyperactivity disorders, ASD and executive deficits [24,25]. In women as well as in men with FXS, the literature reports a strong association between ID and autistic features, with individuals with a lower IQ being more likely to be diagnosed with autism [8-10,12,14,15,20,26]. A negative correlation between IQ scores and the intensity of autistic symptoms, as measured with the ADOS, was observed in men and women [13,15]. The level of cognitive impairment is a major predictor of autistic behavior in FXS, stronger than FMRP levels in lymphocytes [13,27]. These findings would seem to support the suggestion that the occurrence of autistic features in individuals with genetic syndromes and ID is largely the consequence of the cognitive deficit, which decreases their compensatory capacity [28]. Here, we describe the case of two sisters carrying a full mutation of *FMR1* with high-functioning ASD, suggesting that autistic features can emerge independently from ID, at least in women. It should be noted, however, that both our patients have an IQ that is low relative to estimates based on familial intelligence, as shown previously in women with FXS [29]. In agreement with our findings, a previous study found a lack of correlation between autistic behaviors and IQ among girls with FXS [30]. Thus, fragile X mutations can manifest in a wide variety of clinical diagnoses crossing categorical boundaries, specifically ID and ASD. The mechanisms involved in the determinism of specific trajectories remain to be elucidated and could be influenced by genetic, epigenetic and environmental processes [26].

The prevalence of women with high-functioning ASD and FXS is probably underestimated because FXS testing is not systematically included in the etiological screening of patients with high-functioning ASD. In the absence of a family history of ID, premature ovarian failure or Parkinsonism, the search for *FMR1* abnormalities is usually not recommended in patients with high-functioning ASD (American Academy of Pediatrics, [31]). Shyness or social anxiety, commonly mentioned in studies of women with FXS, could be, at least in some cases, misinterpretations of symptoms belonging to the autism spectrum. Also, mild manifestations of ASD are not evaluated or are misevaluated in most reports by the use of inadequate clinical instruments to explore social and communicative deficits in patients with FXS. Thus, if ASD were indeed underestimated in high-functioning patients with FXS, one could suggest that ID and ASD could be phenotypic variants, spanning from ID without ASD to ASD without ID. Specifically in women with FXS, the significant heterogeneity reported for cognitive impairment ranging from severely impaired to normal IQ, or even high IQ in rare cases [32], could also be true for autistic symptoms, with some individuals presenting independence between both dimensions, as shown for many other genetic disorders implicated in the etiology of ASD [33]. In particular, many X-linked ID genes have been associated with a profound phenotypic heterogeneity and can be disrupted in individuals without ID. For example, *NLGN4X* mutations are involved in a wide spectrum of phenotypes, ranging from mild isolated ID without communication deficits to Asperger syndrome with normal intelligence (for review see [34]).

The phenotypic severity of full mutations in women is widely assumed to be determined by X inactivation [18,19]. However, in our patients, the proportion of active normal alleles was approximately at equilibrium (0.55) in both cases. This contrasts with previous findings, in which women who are phenotypically normal have a skewed X inactivation pattern in favor of the inactive X carrying the mutation [18,19]. Additional mechanisms may underlie the variable expressivity observed in FXS. First, the individual genetic background may modulate the FXS phenotype. In our proband, however, the search for rare CNVs did not reveal mutations in other genes that could explain the development of autistic features independently of ID. Second, variations in the FMRP level resulting from mosaicism of the *FMR1* repeat expansion or incomplete *FMR1* promoter methylation [13,35] can also impact the phenotypic variability observed in patients. For example, there are several reports of male patients with FXS with learning disability but not ID, with partial FMRP expression arising from transcription of unmethylated alleles [35], including a male with Asperger syndrome [36]. In the sisters reported here, the mutated alleles were fully methylated. Finally, the partial block in 21-dehydrogenase reported in Patient 1 could potentially explain why she was more severely affected than her younger sister. Girls with congenital adrenal hyperplasia have more autistic traits than their unaffected sisters, suggesting that prenatal exposure to high levels of testosterone increases the vulnerability to ASD [37].

## Conclusions

Our report highlights the necessity for further investigation of the association between ASD and FXS in larger samples of high-functioning female individuals with ASD, which could contribute to the understanding of the complexity of the relationship between ID and ASD. In FXS as well as in many other genetic disorders, patients have been shown to exhibit autistic symptoms spanning from mild to severe, associated or not with ID [33]. This report stresses the importance of clinicians being aware of the association between a full mutation of *FMR1* and high-functioning ASD in women and, vice versa, the importance of a careful psychiatric examination of women with FXS.

## Abbreviations

ADI-R: Autism Diagnostic Interview-Revised; ADOS: Autism Diagnostic Observation Schedule; AR: Androgen receptor; ASD: Autism spectrum disorder; CNV: copy number variation; FMRP: Fragile X mental retardation protein; FXS: Fragile X syndrome; FXTAS: Fragile X-associated tremor/ataxia syndrome; ID: Intellectual disability; IQ: Intellectual quotient; PCR: Polymerase chain reaction; PDD-NOS: Pervasive developmental disorder not otherwise specified; WAIS: Wechsler Adult Intelligence Scale.

## Competing interests

The authors declare they have no competing interests.

## Authors’ contributions

PC, MGB, AB and ML helped to collect the data. CB and RD interpreted the results, drafted the manuscript and were, with ML and TB, the main investigators. SK and SD performed the molecular experiments. All authors read and approved the final manuscript.
